# Neurocognitive Correlates of Approach and Avoidance Behavior in Alcohol-Dependent Patients During a Motivational Monetary Go/No-Go Task

**DOI:** 10.1192/j.eurpsy.2026.10822

**Published:** 2026-07-31

**Authors:** L. M. Rosin, V. Scholz, C. Brosig, R. Maier, M. J. Herrmann, L. Deserno

**Affiliations:** 1 Department of Child and Adolescent Psychiatry, Psychosomatics and Psychotherapy, Centre of Mental Health; 2 Department of Psychiatry, Psychosomatics and Psychotherapy, Centre of Mental Health, University of Würzburg, Würzburg; 3 Department of Psychiatry and Psychotherapy, Technical University Dresden, Dresden; 4 Max-Planck-Institute for Human Cognitive and Brain Sciences, Max Planck, Leipzig, Germany

## Abstract

**Introduction:**

There is growing evidence that motivational biases in alcohol-dependent (AD) individuals may contribute to the persistence of addictive behaviour. One such mechanism is the Pavlovian Bias – the automatic approach of rewards and avoidance of punishment – which can significantly impact intentional choices. However, evidence is still limited on whether such biases extend to non-substance-related contexts.

**Objectives:**

This study aimed to test whether AD patients display stronger Pavlovian Biases than healthy controls (HC), with a specific focus on performance in incongruent conditions such as NoGo-to-Win.

**Methods:**

We investigated 26 AD patients and 26 HC using a motivational monetary Go/No-Go task. Task design orthogonally manipulated action (Go vs. No-Go) and outcome valence (reward vs. punishment), resulting in congruent (Go-to-Win, NoGo-to-Avoid) and incongruent (Go-to-Avoid, NoGo-to-Win) conditions. Accuracy and Go probabilities were analysed across groups and blocks using mixed-effects models.

**Results:**

Across groups, participants showed a Pavlovian Bias with more Go responses in reward trials. In task block 2, AD individuals committed significantly more inappropriate Go responses in No-Go trials than controls, particularly under reward conditions, suggesting stronger approach tendencies and reduced inhibitory control. While healthy controls improved their performance in motivationally incongruent trials over time, patients adapted less. In the second task block, AD individuals were significantly less accurate across conditions.

**Conclusions:**

These findings suggest impaired goal-directed control and heightened automatic response tendencies in alcohol dependence, particularly under motivational conflict. This may reflect reduced feedback sensitivity and impaired cognitive control, pointing to broader mechanisms that sustain maladaptive behaviour beyond substance-specific contexts.

**Disclosure of Interest:**

None Declared

